# Proteomics-Based Systems Biology Modeling of Bovine Germinal Vesicle Stage Oocyte and Cumulus Cell Interaction

**DOI:** 10.1371/journal.pone.0011240

**Published:** 2010-06-21

**Authors:** Divyaswetha Peddinti, Erdogan Memili, Shane C. Burgess

**Affiliations:** 1 Department of Basic Sciences, College of Veterinary Medicine, Mississippi State University, Mississippi State, Mississippi, United States of America; 2 Department of Animal and Dairy Sciences, Mississippi State University, Mississippi State, Mississippi, United States of America; 3 Institute for Digital Biology, Mississippi State University, Mississippi State, Mississippi, United States of America; 4 Life Sciences and Biotechnology Institute, Mississippi State University, Mississippi State, Mississippi, United States of America; 5 Mississippi Agricultural and Forestry Experimental Station, Mississippi State University, Mississippi State, Mississippi, United States of America; Ottawa Hospital Research Institute and University of Ottawa, Canada

## Abstract

**Background:**

Oocytes are the female gametes which establish the program of life after fertilization. Interactions between oocyte and the surrounding cumulus cells at germinal vesicle (GV) stage are considered essential for proper maturation or ‘programming’ of oocytes, which is crucial for normal fertilization and embryonic development. However, despite its importance, little is known about the molecular events and pathways involved in this bidirectional communication.

**Methodology/Principal Findings:**

We used differential detergent fractionation multidimensional protein identification technology (DDF-Mud PIT) on bovine GV oocyte and cumulus cells and identified 811 and 1247 proteins in GV oocyte and cumulus cells, respectively; 371 proteins were significantly differentially expressed between each cell type. Systems biology modeling, which included Gene Ontology (GO) and canonical genetic pathway analysis, showed that cumulus cells have higher expression of proteins involved in cell communication, generation of precursor metabolites and energy, as well as transport than GV oocytes. Our data also suggests a hypothesis that oocytes may depend on the presence of cumulus cells to generate specific cellular signals to coordinate their growth and maturation.

**Conclusions/Significance:**

Systems biology modeling of bovine oocytes and cumulus cells in the context of GO and protein interaction networks identified the signaling pathways associated with the proteins involved in cell-to-cell signaling biological process that may have implications in oocyte competence and maturation. This first comprehensive systems biology modeling of bovine oocytes and cumulus cell proteomes not only provides a foundation for signaling and cell physiology at the GV stage of oocyte development, but are also valuable for comparative studies of other stages of oocyte development at the molecular level.

## Introduction

Germinal vesicle (GV) breakdown is fundamental for maturation of fully grown, developmentally competent mammalian oocytes. Intercellular communication between oocytes and cumulus cells at GV stage is essential for proper maturation or ‘programming’ of oocytes, which is crucial for fertilization and embryonic development [1], [2]. ‘Gap junctions’ in the regions of oocyte and cumulus cells association allow nutrient and paracrine factor transport between oocytes and cumulus cells [2], [3], [4]. Cumulus cell removal before maturation, or the obstruction of gap junctions, suppresses oocyte maturation [5], [6], [7], [8], [9]. Furthermore, cumulus cells are proposed to protect oocytes by preventing oxidative stress-induced cell death and DNA damage by increasing oocyte glutathione content [10] and thus functionally influence oocyte competence. In turn, via secreted factors, oocytes regulate folliculogenesis by promoting: granulosa cell proliferation, differentiation, and gene expression as well as cumulus cell expansion [2], [11]. Folliculogenesis fails in the absence of oocyte paracrine signaling, (whether due to genetic deficiency or experimental oocyte ablation) [2], [12], [13]. Although this oocyte and cumulus cell bidirectional communication is essential for competent oocyte development, the molecular details underlying this communication remain poorly defined. There is thus still a lack of reliable molecular markers and valid definition of a high quality oocyte have impeded the selection of optimal oocytes necessary for assisted reproductive techniques (ARTs) at a high efficiency in humans as well as farm animal species.

Published studies with mouse model show that cumulus cells play an important role in nutritional support of the developing oocyte in the form of pyruvate [14], [15], [16] and stimulation of this nutritional support of cumulus cells is in turn dependent upon the presence of paracrine factors secreted by the oocytes [17]. Although most basic reproductive biology work is done in the mouse [18], significant species differences in oocyte biology exist between humans and mice [19], [20]. The bovine is a relevant animal model for studies of oocyte and cumulus cell communication in human because oocyte biology, and many aspects of ovarian follicular dynamics, is similar between these two single ovulating species [14], [15]. Bovine fertility is also important on its own merit; it has implications in agro-economics involving cattle industry worldwide. Evidences using both the bovine and porcine models show that attachment of cumulus cells to the oocyte during meiotic maturation and fertilization is critical for promoting subsequent embryo development [7], [8], [21], [22].

Proteins primarily determine cell phenotypes and here we used a shotgun proteomics approach that allows us to relatively quantify which proteins are actually expressed in the cell compartments (as opposed to what might be or have the potential to be). This is especially important in oocytes, where there is no linear correlation between amounts of mRNA and the proteins they encode [23]. We previously analyzed the proteomes of bovine germinal vesicle (GV) stage oocytes and their surrounding cumulus cells using differential detergent fractionation two-dimensional liquid chromatography electrospray ionization tandem mass spectrometry (DDF 2D-LC ESI MS/MS) [19]. In the previous study, we reported the first descriptive map of bovine GV oocytes and their potentially-interacting cumulus cells with specific emphasis on membrane, nuclear proteins, receptor-ligand pairs, and transcription factors. Here, and in contrast, using separately-harvested bovine GV oocyte and cumulus cells, we did a separate proteomics experiment but this time using an updated bovine proteome, more stringent search criteria, and much better structural and functional annotation. This has allowed us to do more comprehensive quantitative computational systems biology modeling of oocyte and cumulus cell communication.

Biological systems utilize highly complex, interrelated networks and pathways to function and in contrast to our previous work [19], here we used two complementary computational systems biology modeling approaches: Gene Ontology (GO; [24]) –based and canonical genetic-network-based, to derive understanding from our large dataset. As part of this process, we functionally-annotated all oocyte and cumulus proteins we identified. This improved these protein's GO annotation quality score (GAQ) [25], and enhanced our ability to do GO quantitative modeling to identify the biological processes that are either agonistic or antagonistic to GV oocyte development and which may affect oocyte competence and maturation. Our data also provide a foundation for further structural and functional annotation of the bovine genome specifically-focused on identifying genes associated with developmental competency that could be used for selecting oocytes in manipulating mammalian reproduction. Complementing our biological process modeling, our canonical genetic network modeling identified signaling pathways likely to be involved in the bidirectional communication between oocytes and cumulus cells. Together our analyses can serve as a basis for further “omic” or reductionist research on oocyte and cumulus cell communication and related reproductive abnormalities.

## Results

### GV oocyte and cumulus cell proteomes

**Figure 1:**
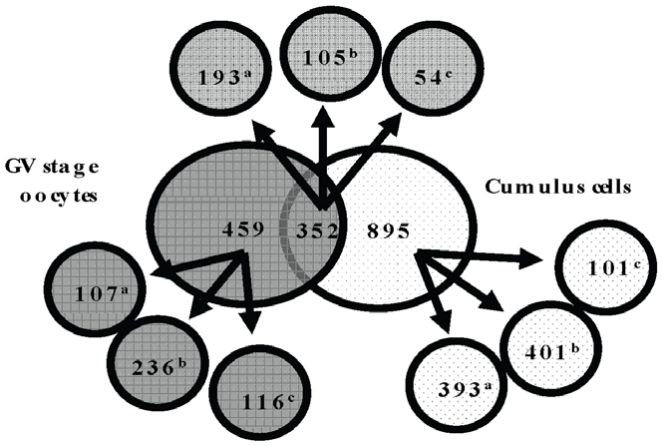
Comparison of proteins identified in germinal vesicle (GV) oocytes and cumulus cells. Distribution of predicted, known, and hypothetical proteins in GV oocytes and cumulus cells is shown. Superscript a, b and c = known, predicted and hypothetical proteins, respectively.

We identified 811 and 1,247 proteins in GV oocyte and cumulus cells, respectively (Table S1). All protein identifications and MS/MS data have been submitted to the PRoteomics IDEntifications database (PRIDE [26]; accession #: 8691, 8692, 8693, 8694, 8695 and 8696, representing the 6 new datasets generated in this work). Of the total 2,058 proteins, 352 (20.4%) were common to both cell types and 459, and 895 were unique to GV oocyte, and cumulus cells, respectively (Figure 1); 371 proteins were differentially-expressed between GV oocyte and cumulus cells (301 proteins had higher and 70 had lower expression in the cumulus cells, compared to the GV oocytes [Table S2]). Only 702 proteins (41.1%) were annotated as ‘known’ and their expression at protein level have been experimentally validated (23% cumulus cell specific, 6.3% GV oocyte specific, and 11.3% common to both [Figure 1]). Approximately 43% of the identified proteins were annotated as ‘predicted’ based on sequence homology and their expression at protein level has not previously been confirmed [27]. Our proteomics data has contributed to the bovine genome annotation by experimentally-confirming the in vivo expression of 742 electronically predicted proteins (Table S1). We also identified 6.5% and 5.7% of ‘hypothetical’ proteins (i.e. proteins predicted from nucleic acid sequences and that have not been shown to exist by experimental protein chemical evidence [28]) specific to GV oocyte and cumulus cells, respectively and 3.2% common to both cell types.

It is complicated to directly compare between the data set in present study with our previous study [19]. First this is because here we used more sophisticated proteomics methods. Secondly we used a bovine RefSeq database [08/28/2007; 25,078 entries] with very many different protein identification numbers (GI numbers) and protein names; previously *Bos taurus*, had no RefSeq database and so we had to derive a non-redundant protein database [NRPD; National Center for Biotechnology Institute. (NCBI); 07/20/2005; 39,963 entries] using search terms ‘Bos’ and ‘taurus’. Together this means that any estimate of overlap based on GI or name will be an underestimate. Here we BLAST searched the protein sequences of the smaller current dataset against the larger previous dataset and found an overlap of 439 (26%) (Figure S1).

### GO Modeling

**Figure 2:**
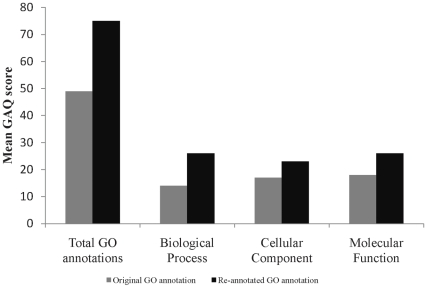
Mean Gene ontology Annotation Quality (GAQ) scores. Mean Gene ontology Annotation Quality (GAQ) score of original and improved Gene onltology annotations of germinal vesicle (GV) oocyte and cumulus cell proteome data sets before and after reannotation.

**Figure 3:**
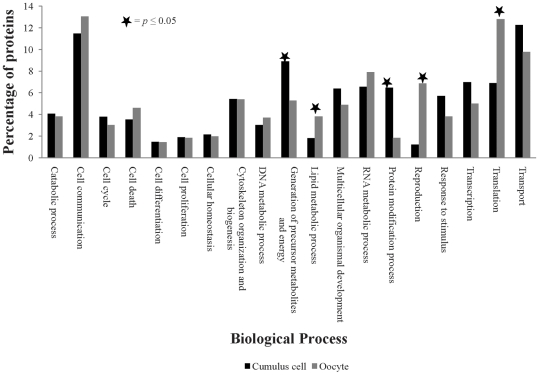
Gene Ontology (GO) modeling of germinal vesicle (GV) oocyte and cumulus cell proteomes. Distribution of percentages of GV stage oocyte and cumulus cell proteins involved in various biological processes. Significant differences in percentage of proteins involved in various identified GO categories in between GV oocyte and cumulus cells were evaluated by student's t-test.

Predicted and hypothetical proteins do not have any functional annotation associated with them and they represent ∼60% of total proteins in our oocyte and cumulus datasets. To compensate for this lack of functional annotation, and to provide the best foundation for biological modeling, we GO-annotated all proteins in our GV oocyte and cumulus data sets. We annotated all the proteins identified in GV oocyte and cumulus cells using GO and obtained annotation for 765 and 1159 proteins of GV oocyte and cumulus cell, respectively. Compared with our previous work, the GAQ score, which is measure of GO annotation quality [25] was almost doubled from 49.2 to 75 (Figure 2). This allowed us to do comprehensive modeling of bovine GV oocyte and cumulus cell communication. Grouping of biological process annotations into more generalized GO categories using a generic GO slim revealed 19 functional categories were represented in GV oocyte and cumulus cell proteomic datasets. Functional categories and percentages of proteins in each category from oocyte and cumulus are shown in Figure 3. The largest GO category represented in GV oocyte was related to cell communication with translation, transport, RNA metabolism, reproduction, and cytoskeletal organization and biogenesis also well represented (42.7% of the oocyte proteome). The largest represented GO category in cumulus cells was transport with cell communication, generation of precursor metabolites and energy, transcription, translation, and protein modification also substantially represented (40.6% of the cumulus cell proteome). Membrane and nuclear proteins are fundamental for inter- and intracellular signaling and are thus fundamental for modeling cell–cell interactions. From the cellular component GO, we identified 404 membrane proteins (24% of the total proteins): 248 unique to CC, 84 unique to oocytes, and 72 in both cell types. Using GO associations, we also identified 273 nuclear proteins: 172 unique to CC, 46 unique to oocytes, and 55 in both cell types (Table S3).

**Figure 4:**
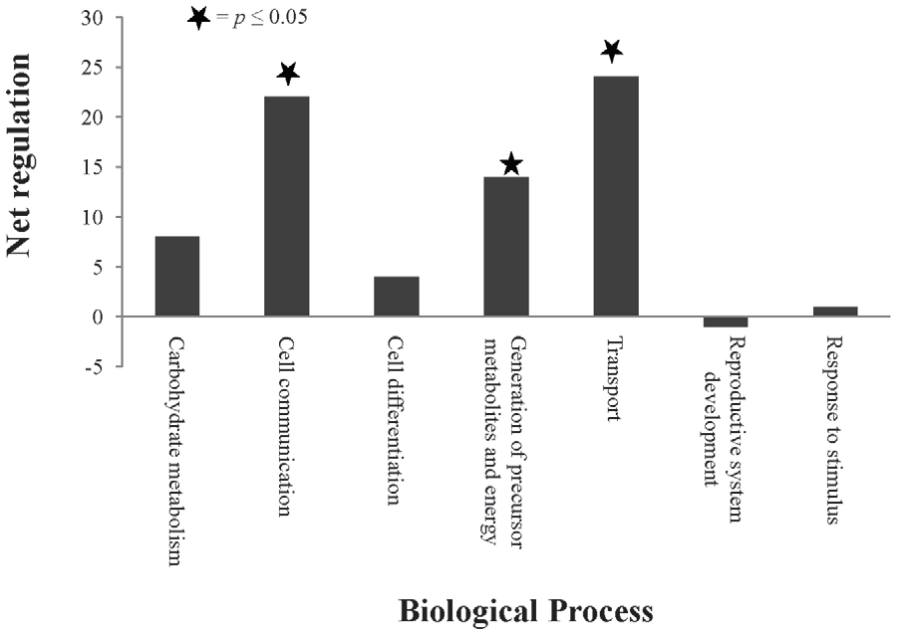
Overall effects in GO Slims of differentially expressed proteins of germinal vesicle (GV) oocyte and cumulus cells. Biological process GO annotations of all differentially-expressed proteins between GV oocyte and cumulus cells were used to generate GO Slims. For each GO Slim the difference in the numbers of proteins with higher expression and the number of proteins with lower expression in cumulus cells (relative to GV oocyte) was calculated to estimate the net regulatory effect.

We next focused on the 371 proteins differentially expressed between GV oocyte and cumulus cells. Application of the AgBase generic GO Slim [29] revealed that 7 functional categories were represented in these differentially expressed proteins. In comparison to oocytes, cumulus cells had significantly higher expression of proteins involved in three biological processes: generation of precursor metabolites and energy, transport, and cell communication (Figure 4).

**Figure 5:**
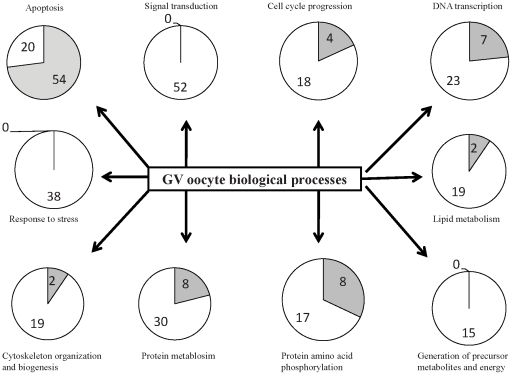
Germinal vesicle (GV) oocyte biological processes. Numbers of proteins agonistic (white) or antagonistic (grey) for each biological process including signal transduction, cell cycle regulation, DNA transcription, apoptosis regulation, protein metabolism and modification, generation of precursor metabolites and energy, cytoskeleton organization and biogenesis, and response to stress and calculated the net effect.

Our GO based quantitative modeling also showed that GV stage oocytes are biased towards biological processes such as cell cycle regulation, signal transduction, DNA transcription, protein metabolism and modification, generation of precursor metabolites and energy, response to oxidative stress, protein amino acid phosphorylation, and cytoskeletol organization and biogenesis but biased against apoptosis (Figure 5).

### Canonical genetic network analysis

Analysis of protein-protein interactions as part of complexes, pathways and biological networks is complimentary to analysis of functional annotations and here we used canonical pathway analysis. Among the 811 proteins identified in GV oocytes, 727 proteins had information about their contribution in canonical networks and functions/pathways. We identified 30 canonical networks and 49 functions. Functions of proteins involved in the top five networks were related to protein synthesis, DNA replication, recombination and repair, cell-to-cell signaling and interaction, molecular transport, amino acid metabolism, reproductive system development and function, small molecule biochemistry, and cellular function and maintenance. Of the 1247 proteins identified in cumulus cells, 1114 had information about their contribution in canonical genetic networks and functions/pathways, respectively. We identified 46 networks and 50 functions. Functions of proteins involved in top five networks were related to cell-to-cell signaling and interaction, molecular transport, protein synthesis, nucleic acid metabolism, cellular function and maintenance, small molecule biochemistry, molecular transport, RNA trafficking and post translational modification. The top five networks (ranked based on statistical significance), and their associated proteins are shown in Table 1 and 2 for GV oocyte and cumulus cell, respectively.

**Table 1:** The top five biological networks in bovine GV oocyte proteome.

Network IDs	Proteins in Networka)	Scoreb)	Focus Proteins	Top Functions
1	*Akt*, **ANGPTL3, C17ORF61, C7ORF20, DDB1, EEF2, EEF1A1, EEF1A2, EEF1B2, EEF1D, EEF1G, ENO1, ENO3**, *Enolase*, **FAM62A, GPI, HARS, HRNR, HYOU1, ILF2 (includes EG:3608), KRT10, PDCD5, PHIP, PLA2G1B, PPT1**, *Protein-synthesizing GTPase*, **RAD23B, RAI14, RPS7, SRGAP1, TSFM (includes EG:10102), TUBB4, TUFM, WARS, YWHAZ**	52	32	Protein synthesis, Lipid metabolism, Small- molecule biochemistry
2	**AHCY, AK2, AURKA, CACYBP, DDX6, EIF4ENIF1, EIF5A, ELAVL1, G3BP1, H2AFX, HIST3H2A, HMGB2, HNRNPK**, *Importin alpha/beta*, *Importin beta*, NME2, **NUP50, PCBP2, PCBP1 (includes EG:5093), PDIA6**, *Pkc(s)*, **RAG1, RALY, RAN, RBMX, RPL23, RPS2, RPS20, TAGLN, TERF1, TGM1, TLN1, UBE2N, UGP2, UQCRC2**	52	32	Molecular transport, Protein trafficking, Amino acid metabolism
3	**ACR**, *Adenosine-tetraphosphatase*, **ALDH2**, *ATP synthase*, **ATP5A1, ATP5B, ATP5C1, ATP5G2, ATP5O**, *ATPase*, **BAT1**, *ERK*, **ETFA (includes EG:2108), ETFB (includes EG:2109), FAU, FHIT**, *GOT*, **GOT2**, *H+-transporting two-sector ATPase*, **HSPD1, HSPE1, M6PRBP1, MAPKAPK3, MFGE8, MVP, NSF, PJA1, RAB1A, RAB2A, RAB7A, RABEP1**, *Rsk*, **STX12, ZP2, ZP3**	42	28	Cell-to-cell signaling and interaction, Reproductive system development and function, DNA replication, recombination, and repair
4	*26s Proteasome*, **AGA, COX5B, DNAJA1, DNMT1, EPHA2, FKBP4**, *HSP*, *Hsp70*, *Hsp90*, **HSP90AA1, HSP90AB1, HSPA2, HSPA5, HSPA6, HSPA8, HSPA9, HSPB1, HSPH1, IARS2, MDH1, MTHFD1**, *Nos*, **PARK7, PARP1, PFN1, PGK1**, *PI3K*, **PPA1, PSAP, PTGES3 (includes EG:10728), RPL7, RPS3A**, *Shc*, **STIP1**	*42*	28	Cellular function and maintenance, Cellular compromise
5	**AICDA, Ant, CYCS (includes EG:54205)**, *Cytochrome c oxidase*, *DDOST*, *Dolichyl-diphosphooligosaccharide-protein glycotransferase*, *Glutathione peroxidase*, **GPX1, IL1F5, INPP1**, *Ldh*, **LDHA, LDHB**, *NF-kappaB (family)*, *NFkB (complex)*, **PECAM1**, *peroxidase (miscellaneous)*, **PRDX1, PRDX2, PRDX3, PRDX4, PRDX5, PRDX6, RAB3C, RPN1, RPN2, SLC25A3, SLC25A4, SLC25A10, SLC25A13**, *Sod*, **SOD2, TRPM8, TXN, VDAC1**	38	26	Small molecule biochemistry, Molecular transport, Cellular function and maintenance

a) The focus proteins are indicated with gene names and shown in bold letters.

b) A score of >2 is considered statistically significant.

**Table 2:** The top five biological networks in bovine cumulus cell proteome.

Network IDs	Proteins in Networka)	Scoreb)	Focus Proteins	Top Functions
**1**	**ACADM, ATP1A2, BCKDHA, C7ORF20, CAPNS1, CSPG4, DPYSL2**, *Enolase*, *ERK*, **ETFA (includes EG:2108), ETFB (includes EG:2109), FAU, FHIT, GAK, HSPE1, IPO7, LGI1, MAP1B, MFGE8, MPZL1, MVP, NUMA1, PELP1, POSTN, PRKCSH**, *Rab11*, **RAB11B, RAB1A, TUBA3C, TUBA4A, TUBB2C**, *Tubulin*, ULBP3, ZP2, ZP3	43	31	Cell-To-Cell Signaling and Interaction, Reproductive system development and function
**2**	**ACTR6, AK2, ANXA4, AP3D1, BRP44, CALU, EEF2, EEF1A1, EEF1A2, EEF1B2, EEF1D, FOS**, *Immunoproteasome Pa28/20s*, **NIPSNAP1, OPTN**, *Proteasome PA700/20s*, *Protein-synthesizing GTPase*, ***PSMA*** **, PSMA1, PSMA2, PSMA4, PSMA6, PSMA7, PSMB2, PSMB4, PSME1, PSME2, PTPRN, RPLP1, RPLP2, RPLP0 (includes EG:6175), RPS18, SEC23B, TKT, TUFM**	41	31	Protein synthesis, Molecular transport, Nucleic acid metabolism
**3**	*Adaptor protein 2*, *Ap1*, **AP1G1, BLVRA, BTF3, CD58**, *Ck2*, *Clathrin*, **CLTC, CSNK2A1, EIF5A, FKBP3, FKBP10, GTF2F1, HNRNPA2B1, HNRNPL, IGF2R, NUCKS1**, *Peptidylprolyl isomerase*, *Phosphatidylinositol4,5 kinase*, **PIN1, PIP4K2A, POLR3C, PPIB, PSMA3, RGS19, RPL5 (includes EG:6125), SSB (includes EG:6741), TF, TFR2, TFRC, TOP1, TUBB4, VPS35, WARS**	38	29	Cellular function and maintenance, Small molecule biochemistry, Molecular transport
**4**	*3-hydroxyacyl-CoA dehydrogenase*, **ACAT1**, *Acetyl-CoA C-acetyltransferas* ***e***, **ATIC, B4GALT1, CYLD, DAD1, DDOST**, *Dolichyl-diphosphooligosaccharide-protein glycotransferase*, **ECH1, ECHS1**, *Enoyl-CoA hydratase*, **ENPP1**, *GOT*, **GOT2, HADHA, HADHB, HSD17B4, HSD17B10**, *NFkB (complex)*, **NME2, OSTC, PDIA6, PGRMC1**, *PPARα-RXRα*, **PRDX4, RPN1, RPN2, SFXN1, SLC27A1, STK10, STT3A, TOMM20, TOMM40, TOMM70A**	36	28	Lipid metabolism, Small molecule biochemistry, Post-translational modification
**5**	**ALPL, ANP32B, C21ORF33, CBX2, CCND1, CCT8, CDC37, DDX3X, DHRS12, EIF3D, EIF4A2, EIF4ENIF1, ELAVL1, ENO3, H1F0, H3F3B**, *Hdac*, *Histone h3*, *Histone h4*, **HNRNPM**, *Importin beta*, **INHA, KPNB1**, *Mi2*, **NCAPD2, PNO1, RAN, SF3B1, SF3B3**, *SWI-SNF*, *Tcf/lef*, **THRAP3, TNPO1, VRK1, XPO1**	36	28	Molecular Transport, RNA Trafficking

a) The focus proteins are indicated with gene names and shown in bold letters.

b) A score of >2 is considered statistically significant.

Twenty-three and 39 canonical pathways were significantly represented in GV oocytes and cumulus cells, respectively. Oxidative phosphorylation is the top represented canonical pathway significant only in cumulus cells. In this pathway, expression of 21 proteins was significantly altered and all these proteins have higher expression in cumulus cells compared to GV oocyte. These proteins include 9 ATP (adenosine triphosphate) synthases (ATP5A1, ATP5B, ATP5C1, ATP5F1, ATP5H, ATP5J, ATP5L, ATP5O, and ATP6V1E1), 3 cytochrome-c-oxidases (COX2, COX17, and COX5A), cytochrome C-1(CYC1), 6 NADH (reduced nicotinamide adenine dinucleotide) dehydrogenase (ubiquinone) complexes (NDUFA5, NDUFA9, NDUFB, NDUFC2, NDUFS2, and NDUFV2) and 2 ubiquinol cytochrome c reductase core proteins (UQCRC1 and UQCRC2) (Table S2). Glycolysis and pyruvate metabolism pathways were significantly represented in both GV oocyte and cumulus cell proteomes. Expression of 12 key enzymes involved in glycolysis/pyruvate metabolism, including triosephosphate isomerase (TPIS), enolase 1 and 2 (ENO1 and ENO2), pyruvate kinase muscle (PKM), fructose-bisphosphate aldolase A (ALDOA), fructose-bisphosphate aldolase C (ALDOC), lactate dehydrogenase A (LDHA), phosphoglycerate mutase 1 (PGAM1), phosphoglycerate kinase 1 and 2(PGK1 and PGK2), glucose phosphate isomerise (GPI), and aldehyde dehydrogenase 3 family member A2 (ALDH3A2) was higher in cumulus cells compared to oocyte (Table S2).

### Cell-to-cell signaling network

**Figure 6:**
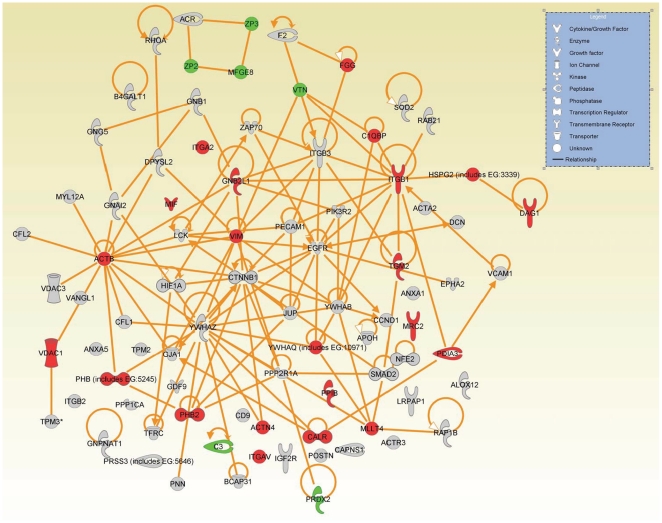
Cell-to-cell signaling and interaction network. Network generated with proteins involved in cell-to-cell signaling biological process using Ingenuity Pathway Analysis (IPA) as described in materials and methods. Each node represents a protein; proteins in shaded nodes were found in either GV oocyte or cumulus cell or both (see Table 3). Proteins in red and green nodes were higher and lower, respectively, in cumulus cells compared with GV oocytes.

**Figure 7:**
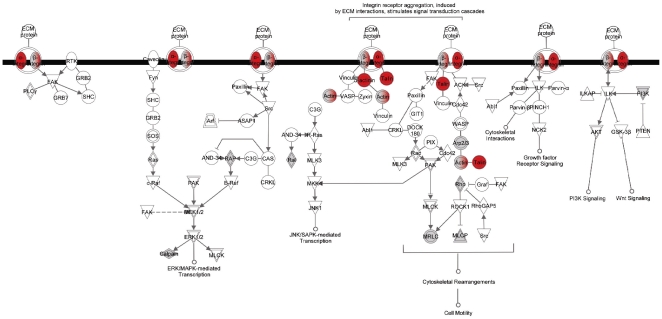
Integrin signaling pathway Integrin signaling pathway generated by the Ingenuity Pathway Analysis (IPA) software. Integrin and actin cytoskeleton signaling pathways were the top two pathways associated with cell-to-cell signaling. Each node represents a protein; the proteins in shaded nodes in the pathway are identified/relate to an identified protein in the proteomic analysis. While proteins in clear nodes are part of the pathway but have not been identified in the GV oocyte or cumulus datasets. Proteins in red nodes were shown higher expression in cumulus cells compared to GV oocyte.

Bidirectional communication between oocytes and cumulus cells is essential for the development and function of both cell types [1], [11], [15]. We focused on the 91 identified proteins GO-annotated as involved in cell-to-cell signaling; 29 of these proteins were differentially-expressed between GV oocyte and cumulus cells (23 were greater and 6 lower in the cumulus cells compared to GV oocytes [Table S4]). The canonical network generated by IPA using proteins involved in cell-to-cell signaling is shown in Figure 6. We next overlaid canonical pathway information on to this network to identify important signaling pathways involved in the intricate cross talk between the GV oocyte and its surrounding cumulus cells. Major pathways associated with proteins involved in this cell-to-cell signaling network are related to integrin signaling, actin cytoskeleton signaling, mitogen-activated protein kinase (MAPK) signaling, phosphoinositide 3-kinase (PI3K) signaling, and ephrin receptor signaling (Table 3). Expression of six proteins– actin, beta (ACTB), actinin, alpha 4 (ACTN4), integrin alpha 2(ITGA2), integrin alpha V (ITGAV), integrin beta 1 (ITGB1), and talin 1(TLN1)—involved in integrin signaling and actin cytoskeleton signaling was higher in cumulus cells compared to GV oocyte (Figure 7) (Table S4). Expression of six proteins—zona pellucida glycoprotein 2[ZP2], zona pellucida 3[ZP3], periredoxin 2[PRDX2], complement component 3[C3], milk fat globule-EGF factor 8 protein [MFGE8] and vitronectin [VTN]—involved in cell-to-cell signaling was significantly higher in GV oocyte compared to cumulus cells (Figure 6; Table S4).

**Table 3:** Top five signaling pathways associated with cell to cell signaling network.

Pathway name^A^	# of focus proteins	Focus proteins^B^	P-value
Integrin signaling	15	ACTA2, ↑ACTB, ↑ACTN4, ACTR3, CAPNS1, ↑ITGA2, ITGB2, ↑ITGAV,	1.01E-12
		↑ITGB1, ITGB3, MYL12A, PIK3R2,	
		PPP1CA, RAP1B, RHOA, ↑TLN	
Actin cytoskeleton signaling	13	ACTA2, ↑ACTB, ↑ACTN4, ACTR3, CFL1	5.48E-10
		CFL2, F2, ↑ITGA2, ↑ITGB1, MYL12A,	
		PIK3R2, PPP1CA, RHOA	
Ephrin receptor signaling	12	ACTR3, CFL1, CFL2, EPHA2, GNAI2,	1.11E-09
		GNB1, ↑GNB2L1, GNG5, ↑ITGA2,	
		↑ITGB1, RAP1B, RHOA,	
PI3K signaling	9	CCND1, CTNNB1, ↑ITGA2, ↑ITGB1, PIK3R2 PPP2RIA, YWHAB,	5.44E-08
		↑YWHAQ, YWHAZ	
MAPK signaling	9	↑ITGA2, ↑ITGB1, PIK3R2, PPP2RIA, PPP1CA, RAP1B, YWHAB,	2.09E-06
		↑YWHAQ, YWHAZ	

A MAPK : Mitogen activated protein kinase; PI3K ; Phosphoinositide 3-kinase.

B ↑ higher expression in cumulus cells compared to GV oocyte.

## Discussion

Here we provide a foundational, proteomics-based descriptive computational systems-biology modeling of oocyte and cumulus cell interaction at the GV stage. Bidirectional communication between oocytes and cumulus cells is considered essential for proper maturation or ‘programming’ of oocytes, which is crucial for normal fertilization and embryonic development [15]. Cumulus cells are unique in that they are differentiated somatic cells essential for development of a competent oocyte. In turn, oocytes through the secretion of secreted factors (OSF's), regulate a multitude of key cumulus cell functions, which may in turn produce positive regulatory factors that pass back to the oocyte, improving subsequent development [17]. However, despite their importance, little is known about the molecular events and pathways involved in the bidirectional communication between oocyte and cumulus cells. The proteins identified in this study in combination with the functional modeling using GO and IPA can serve as a basis for future hypothesis-driven research on follicle development, oocyte and cumulus cell communication, oocyte maturation, and related reproductive abnormalities.

In this study, we identified 811, and 1247 proteins of oocyte, and cumulus cells respectively, which is a significant increase in information compared to other female gametal proteomic studies [30], [31], [32], [33], [34], [35]. Like all proteomics studies, we identified relatively small number of proteins compared to the numbers of mRNAs that can be identified in transcriptome studies. However, mRNAs are at least one step removed from phenotype; proteins are the functional molecules of cells and there is, in general, a very low correlation between amounts of mRNAs and amounts of the encoded protein [36], [37], [38], [39]. Together this means that mRNA is a much less accurate predictor of phenotype than is protein. Even though the coverage of genetic pathways may be lower from proteomics experiments than transcriptomics experiments, because of the statistics that underlie the pathway analysis we are still able to identify relevant pathways that are important to the phenotype.

This current work is based on a separate previous proteomics study [19] which was smaller and generated less than one third of the data than this study did and did not use tissue replicates. It also uses a newer version of the bovine proteome. Though both studies follow accepted standards in the field, there are some significant technical differences between this and the previous study which exemplify the maturation of the field of proteomics. In this present study, protein identification is more stringent and we used the probabilistic approach of decoy database searching [40]. Decoy database searching is based on the real data and uses a decoy database derived from this real database; as this maintains the same amino acid composition and the search space characteristics. Not only does this method allow a probability estimation for every peptide identification is a false positive (and thus protein) but also, because it uses mass spectra generated in the experiment, it does not rely on arbitrary cut off values derived from unrelated experiments [40]. Here we have more confident protein identifications than in the previous work, though we are likely to have increased our type II error. Even though we analyzed almost three times as many mass spectra in this new study, we ended up “confidently” identifying fewer proteins compared to previous study. In the previous study, we identified 5253 proteins were expressed by the cumulus cells and 1950 proteins were expressed by GV oocytes; in this study, we identified 1247 proteins in cumulus cells, and 811 proteins in GV oocytes.

Maternal effect proteins are the proteins that are present in the oocyte, remain expressed throughout oocyte maturation and early embryonic development until maternal-to-embryonic transition (MET) and they may also be required for successful activation of the embryonic genome. We identified 11 maternal effect proteins in bovine oocytes: Maternal Antigen that Embryos Require (Mater; a.k.a NACHT, leucine rich repeat and PYD containing 5, *Nalp5*,), zona pellucid proteins-2, 3 and 4 (ZP-2, -3, and -4), growth differentiation factor 9 (GDF 9), beta-actin (ACTB), heat shock protein-70 (HSP-70), peroxiredoxins 1 and 2 (PRDX-1, -2), DNA (cytosine-5) methyl transferase one (DNMT1), and fibroblast growth factor-8 (FGF-8) all of which are important to oogensis and early embryonic development [41], [42], [43], [44], [45], [46].

Our previous publication described an initial GO-based functional analysis of bovine GV oocyte and cumulus cells using ‘known proteins’ and focusing on the cellular component Gene Ontology to identify membrane and nuclear proteins. Here, we take a much more comprehensive approach to include not only ‘known’ but also ‘predicted’ and ‘hypothetical’ proteins. By definition, predicted and hypothetical proteins do not have any functional annotation associated with them and they represent ∼60% of total proteins in our oocyte and cumulus datasets. Hence we manually annotated all these predicted and hypothetical proteins based on sequence similarity and orthology from human, mouse and rat proteins. Overall our approach, while much more laborious and requiring recognized GO biocuration skills, provides more comprehensive information on the biological processes associated with bovine GV oocyte and cumulus cell function. Not only does this improved data quality allow a much better modeling, but we almost doubled the Gene ontology Annotation Quality (GAQ) score [25] (a quantitative measure of the GO annotation) of the proteins in our dataset and this will be valuable for others (the annotations are available at the AgBase database ([29]; www.agbase.msstate.edu). By manual literature biocuration, we also captured the quantitative positive and negative regulatory roles that these GV oocyte proteins play in the various biological processes. Overall we could identify and quantify proteins agonistic or antagonistic to biological processes including signal transduction, cell cycle regulation, DNA transcription, apoptosis regulation, protein metabolism and modification, generation of precursor metabolites and energy, cytoskeleton organization and biogenesis, and response to stress in GV oocyte; all of which may have putative roles in oocyte competence and maturation. This type of quantitative modeling is not possible with the available pathway analysis tools. Furthermore, we have analyzed the protein datasets identified in this experiment in the context of protein interaction networks and pathways using Ingenuity Pathway Analysis. This pathway and network modeling also reveals signaling pathways associated with the proteins involved in cell-to-cell signaling biological process which may have implications in various reproductive processes such as oocyte development, and maturation.

Our GO-based modeling identified that the percentage of proteins involved in translation in GV oocytes is twice as high as in cumulus cells and this is consistent with other's data showing that GV oocytes are transcriptionally and translationally active and also that proteins synthesized in this stage might be crucial to achieve maturation and successful subsequent development [19], [47]. We identified a comparative up regulation of three biological processes—generation of precursor metabolites and energy, transport, and cell communication—in cumulus cells compared to oocytes. Up regulation of generation of precursor metabolites and energy in cumulus cells is consistent with a model that compensates for oocytes inability to metabolize glucose in which surrounding cumulus cells may absorb and metabolize glucose to provide products that can be utilized by oocytes for energy metabolism [48], [49]. In support, cumulus cells nutritionally-support the developing oocytes by providing pyruvate [14], [15], [16]. Furthermore, stimulation of this nutritional support is in turn dependent on the presence of paracrine factors secreted by the oocyte (oocyte secreted factors (OSFs)); OSFs stimulate the glycolytic activity in cumulus cells by promoting the expression of genes involved in glycolysis [17] in mice, and our bovine model also suggests the glycolytic support of cumulus cells by showing higher expression of twelve proteins involved in glycolysis in cumulus cells associated with GV oocyte. Glycolysis is the metabolic pathway that converts glucose into pyruvate and the free energy released in this process is used to form the high energy compounds, ATP and NADH. We also identified oxidative phosphorylation as the most prominent metabolic pathway significantly represented only in cumulus cells, but not in oocyte suggesting a hypothesis that cumulus cells may also provide energy to oocytes in the form of ATP. Expression of core proteins (ATP synthases, cytochrome oxidases, NADH dehydrogenases, and ubiquinol cytochrome c reductase) involved in oxidative phosphorylaton was higher in cumulus cells compared to oocytes. NADH dehydrogenases and cytochrome C oxidases are, respectively, the first and last enzymes of the mitochondrial electron transport chain helping to establish a transmembrane difference of proton electrochemical potential that the ATP synthase then uses to synthesize ATP [50].

Cumulus cells provide a network of gap junction transmembrane channels facilitating two way communications for nutrient or paracrine factor exchange between oocytes and cumulus cells [1], [3]. Several small metabolites (such as some energy substrates, nucleotides, and amino acids) are absorbed by oocytes mostly, or entirely, from surrounding cumulus cells via gap junctions [15], [51]. Our bovine model also demonstrated an increase in transport in cumulus cells and this is in agreement with previous studies that cumulus cells have key role in amino acid and energy substrate uptake and transport of these substances to the oocyte.

Cumulus cells are essential for protecting oocytes from oxidative stress-induced apoptosis and DNA damage and their communication with oocytes is essential for development of an oocyte competent to undergo fertilization and embryogenesis [5], [52]. We identified 91 proteins involved in cell-to-cell signaling from oocytes and cumulus cells. To effect intercellular communication several signaling pathways are necessary. We identified the integrin and actin cytoskeleton signaling pathways as the top two associated pathways with cell-to-cell signaling. Integrin signaling converges on cell cycle regulation, directing cells to live or die, to proliferate or to exit the cell cycle and differentiate [53]. Expression of ACTB, ACTN4, ITGAV, ITGA2, ITGB1, and TLN1, involved in integrin signaling and actin cytoskeleton signaling was higher in cumulus cells compared to oocytes. These proteins are the cell surface receptors present in the follicular basement membrane and around follicular cells and participate in cell attachment to matrix and mediate mechanical and chemical signals from it [54]. These signals in turn regulate the activities of cytoplasmic kinases, growth factor receptors, and ion channels that control the organization of the intracellular actin cytoskeleton [53], [54]. The actin cytoskeleton of the human oocyte plays a key role in cell surface morphology as well processes associated with oocyte maturation [55]. Together this data suggests a hypothesis that oocytes depend on the presence of cumulus cells to generate specific cellular signals to coordinate their growth and maturation.

In addition to integrin and actin cytoskeleton signaling pathways, we also identified MAPK signaling, PI3K signaling, and ephrin receptor signaling pathways may involved in bidirectional oocyte-cumulus cell communication. MAPK and PI3K signaling are involved in activation of several membrane signaling molecules followed by sequential activation of protein kinases which involves highly regulated and modulated cascades of phosphorylation events [56]. Protein phosphorylation during this signaling plays a major role in the oocyte meiotic maturation and many protein kinases activated during oocyte maturation are also involved in cumulus cell proliferation and differentiation [57]. Expression of three proteins ITGA2, ITGB, and YWHAQ involved in MAPK and PI3K signaling was higher in cumulus cells compared to oocytes. YWHAQ, a protein of tyrosine 3-monooxygenase/tryptophan 5- monooxygenase activation protein family, modulate/complement intracellular events involving phosphorylation dependent switching or protein modification in mammalian oocytes. The YWHAQ protein has been shown to protect phosphorylated proteins from inopportune dephosphorylation [58]. This is suggesting a hypothesis that cumulus cells have important role in oocyte maturation by regulating the protein phosphorylation, an important event during mammalian oocyte maturation.

We also observed expression of peroxiredoxins (PRDX's) involved in cell-to-cell signaling was significantly higher in oocytes compared to cumulus cells. PRDX's are peroxidases involved in antioxidant defence and intracellular signaling. Cumulus cells induce PRDX up regulation in oocytes [59] and our work also suggests that cumulus cells may have a key role in protecting oocytes from oxidative stress induced apoptosis and DNA damage via inducing oocytes to produce peroxiredoxins.

In conclusion, our systems biology modeling of bovine oocyte and cumulus proteomes, in the context of gene ontology and canonical protein interaction networks identified ninty-one proteins involved in the cell-to-cell signaling biological process that may have role in bidirectional communication between oocytes and cumulus cells. Most of the proteins involved in the cell-to-cell signaling biological process are the components of integrin, actin cytoskeleton, MAPK and PI3K signaling pathways that may have implications in various reproductive processes such as oocyte development, and maturation. In addition, GO-based analysis of differentially expressed proteins facilitated the identification that compared to GV oocytes, cumulus cells have higher expression of proteins involved in the generation of precursor metabolites and energy, transport, and cell communication. This systems biology model of bovine oocytes and cumulus cell potential interacting proteomes not only provides a foundation for understanding signaling and cell physiology at the GV stage of oocyte development, but is also valuable for comparative studies of other stages of oocyte development at the molecular level. The proteomes and biological process identified in this study can also serve as reference points for further comparative studies on immature and abnormal oocyte and cumulus cells elucidating the underlying molecular mechanisms involved in normal and pathological oocyte-cumulus cell communication. Furthermore, some of the proteins involved in cell-to-cell signaling may have value as molecular biomarkers which could be useful for assessing oocyte quality.

## Materials and Methods

### GV oocytes and cumulus cells

Separate from our previous work [19], ovaries from Holstein cows were collected by Biomed Inc. (Madison, WI, USA) from a local slaughter house. Cumulus–oocyte complexes (COC) were aspirated only from the follicles with a diameter of 2–8 mm using an 18-gauge needle attached to a vacuum system [60]. Only oocytes with intact cumulus cell layers and homogenously granulated cytoplasm were selected, washed three times in TL-HEPES supplemented with polyvinylpyrrolidone (3 mg/ml polyvinyl-pyrroline-40; Sigma), Na-pyruvate (0.2 mM) and gentamycin (25 µg/ml). To obtain oocytes free of cumulus cells, COCs were vortexed in TL-HEPES (3 min), oocytes were collected under a stereomicroscope, further vortexed with hyaluronidase to remove adhering cumulus cells completely (3 min), washed thrice in saline and stored in a cell lysis buffer (4°C) until use. The cumulus cells separated from the oocytes after the first vortex were centrifuged, washed twice with saline, and the resulted pellets were resuspended in the lysis buffer and stored (4°C) until use.

### Protein extraction using differential detergent fractionation (DDF)

DDF sequentially extracts proteins from different cellular compartments using a series of detergents and provides information on cell location as well as increasing proteome coverage. Three replicates of five hundred GV oocytes and their surrounding CC were each subjected to DDF exactly as previously described [61]. Briefly, cytosolic proteins were extracted by six sequential incubations in a buffer containing digitonin (10 min each); next a fraction containing predominantly membrane proteins was isolated by incubating the cells in 10% Triton X-100 buffer for 30 min and then removing the soluble protein. Nuclear DDF buffer containing deoxycholate (DOC) was then added to the remaining pellet and subjected to freeze-thawing to disrupt the intact nuclear membrane. Nuclear proteins were collected from the resulting soluble fraction and the sample was then aspirated through an 18-guage needle and treated with a mixture of DNase I (50U, Invitrogen, Carlsbad, CA) and RNase A (50 mg, Sigma-Aldrich, St Louis, MO) at 37°C for 1 h to digest nucleic acids. The remaining undissolved pellet was then treated with a buffer containing 5% SDS.

### Proteomics

Protein quantification and trypsin digestion of DDF fractions were done exactly as described [19]. Briefly, proteins were precipitated with 25% tricholoroacetic acid to remove salts and detergents, then resuspended in 0.1 M ammonium bicarbonate with 5% HPLC grade acetonitrile (ACN), reduced (Dithiothreitol, 5mM, 65°C, 5 min), alkylated (iodoacetamide, 10 mM, 30°C, 30 min) and then trypsin digested until there was no visible pellet (sequencing grade modified trypsin, Promega; 1∶50 w/w 37°C, 16 h). Peptides were desalted using a peptide macrotrap (Michrom BioResources, Inc., Auburn, CA) and eluted using a 0.1% trifluoroacetic acid, 95% ACN solution. Desalted peptides were dried in a vacuum centrifuge and resuspended in 20µL of 0.1% formic acid and 5% ACN.

Two-dimensional liquid chromatography (LC) analysis was accomplished by strong cation exchange (SCX) followed by reverse phase (RP) coupled directly in line with an electrospray ionization (ESI) ion trap tandem mass spectrometer (LCQ; ThermoElectron Corp., San Jose, Calif, USA) essentially as described in [19]. The salt gradient applied in this study was different from our previous published method and was applied in steps of 0, 10, 15, 20, 25, 30, 35, 40, 45, 50, 57, 64, 90, and 700 mM ammonium acetate in 5% acetonitrile (ACN) and 0.1% formic acid. The reverse phase gradient used 0.1% formic acid in ACN and increased the ACN concentration in a linear gradient from 5% to 30% in 20 minutes and then 30% to 95% in 7 minutes, followed by 5% for 10 minutes for 0, 10, 15, 25, 30, 45, 64, 90, and 700 mM salt gradient steps. For 20, 35, 40, 50, and 57 mM salt gradient steps, ACN concentration was increased in a linear gradient from 5% to 40% in 65 minutes, 95% for 15 minutes, and 5% for 20 minutes.

Mass spectra and tandem mass spectra were searched against an *in silico* trypsin-digested bovine database using TurboSEQUEST™ (Bioworks Browser 3.2; ThermoElectron). The bovine database used in this study was different from the previous publication and here we used database of bovine Reference Sequence (RefSeq) proteins downloaded from the National Center for Biotechnology Institute [NCBI; 08/28/2007; 25,078 entries]. The Reference Sequence (RefSeq) collection aims to provide a comprehensive, integrated, non-redundant, and well-annotated set of sequences of proteins [62]. Trypsin digestion including mass changes due to cysteine carbamidomethylation (C, 57.02 Da) and methionine mono- and di-oxidation (15.99 Da and 32 Da), were included in the search criteria. The peptide (MS precursor ion) mass tolerance was set to 1.5 Da and the fragment ion (MS^2^) mass tolerance was set to 1.0 Da. Rsp Value less than 5.

As a primary filter we first limited our Sequest search output to include only peptides ≥6 amino acids long, with ΔCn ≥0.1 and Sequest cross correlation (Xcorr) scores of 1.9, 2.2, and 3.7 for +1, +2, and +3 charge states, respectively. We next used a decoy database search strategy [40] (using the same search criteria as the real database search) to calculate p-values for peptide identifications as this allows us to assign the probability of a false identification based on the real data from the experiment itself [40], [63], [64]. Since the accuracy of peptide identification depends on the charge state we calculated P-values for +1, +2, and +3 charge states separately. The probability that peptide identification from the original database is a random match (P-value) is estimated based on the probability that a match against the decoy database will achieve the same Xcorr [65], [66]. Protein probabilities were calculated exactly as described [67], [68] and we used only proteins identified by peptides with a p<0.05 for further analysis. All protein identifications and MS/MS data have been submitted to the PRoteomics IDEntifications database (PRIDE [26]; accession numbers 8691, 8692, 8693, 8694, 8695 and 8696). PRIDE submission requirements are based on the proposed guidelines by proteomics standards initiative [69] and include all the peptides identified for each protein with their sequence, charge state, Xcorr, and delta cn.

We used an isotope-free quantification method [70] and a custom program *ProtQuant* [71] to identify differences in protein expression between oocyte and cumulus cell datasets. *ProtQuant* is a java based tool for label-free quantification that uses a spectral counting method with increased specificity (and thus decreased false positive i.e. type I errors). This increased specificity is achieved by incorporating the quantitative aspects of the Sequest cross correlation (XCorr) into the spectral counting method. *ProtQuant* also computes the statistical significance of differential expression by one-way ANOVA (α≤0.05).

### Gene Ontology (GO) Annotation

To identify the biological processes of all proteins in our datasets we used Gene Ontology annotations and the GO resources and tools available at AgBase [29]. We had to overcome the limitation that most literature for any species is not yet curated and so functional annotations from this literature are not yet available at GO databases. First, we used *GORetriver* to obtain all existing GO annotations available for known proteins in our datasets [72]. To obtain GO annotations for proteins without existing annotation, but between 70–90% amino acid sequence identity to presumptive orthologs with GO annotation were annotated using GO*anna* by manually checking the similarity between our proteins and orthologs [72]. GO biological process annotations for proteins were grouped into more generalized categories using *GOSlim viewer* [72]. Significant differences in percentage of proteins involved in various identified GO categories in between GV oocyte and cumulus cells were evaluated by *student's t*-*test*. Differences at *p*<0.05 were considered statistically significant.

GO annotation quality (*GAQ*) scores were calculated exactly as described [25] to quantify the improvement in GO annotation quality of GV oocyte and cumulus proteins due to re-annotation in this study compared to existing annotations. The *GAQ* score is a quantitative measure of the GO annotation of a set of gene products based on the number of GO annotations available, the level of detail of the annotation and the types of evidence used to make these GO annotations.

### GO based quantitative modeling

Our GO based quantitative modeling of GV stage oocyte was based on specific hypothesis framed in GO biological process (GOBP) terms defining the phenotype of oocyte competence and maturation. Although GOBP terms exists for gene products that effect and affect oocyte competence and maturation, and there is functional literature on these genes, the literature and GO are unconnected [42]. To connect the data with the GO, we manually annotated the literature of GV oocyte proteins to compare the number of proteins that were either agonistic or antagonistic for each biological process including signal transduction, cell cycle regulation, DNA transcription, apoptosis regulation, protein metabolism and modification, generation of precursor metabolites and energy, cytoskeleton organization and biogenesis, and response to stress and calculated the net effect of each process in GV oocyte; all of which may have a putative role in oocyte competence and maturation.

### Modeling using Ingenuity pathway analysis

To gain insights into the biological pathways and networks that are statistically significantly represented in our proteomic datasets we used Ingenuity Pathways Analysis (IPA; Ingenuity Systems, California). We imported protein accessions from our GV oocyte and cumulus cell datasets into IPA. IPA selects “focus proteins” to be used for generating biological networks. Focus proteins are the proteins from our datasets that are mapped to corresponding gene objects in the Ingenuity Pathways Knowledgebase (IPKB) and are known to interact with other proteins based on published, peer reviewed content in the IPKB. Based on these interactions IPA builds networks with a size of no more than 35 genes or proteins. A p-value for each network and canonical pathway is calculated according to the fit of the user's set of significant genes/proteins. IPA computes a score for each network from the p-value that indicates the likelihood of the focus proteins in a network being found together due to chance. We selected only networks scoring ≥2, which have >99% confidence of not being generated by chance [73] [74]. Biological functions are assigned to each network by using annotations from scientific literature and stored in the IPKB. A Fisher exact test is used to calculate the p-value determining the probability of each biological function/disease or pathway being assigned by chance. We used P≤0.05 to select highly significant biological functions and pathways represented in our proteomic datasets [73].

## Supporting Information

Figure S1 Comparison of proteins identified in present study with previous published study by Memili et al., 2007.
(0.01 MB PDF)

Table S1 List of proteins identified in germinal vesicle (GV) stage oocyte and cumulus cells. Proteins identified by DDF-MudPIT and their distribution in GV oocyte and cumulus cells. GI numbers of the identified proteins (column A) and corresponding protein names (assigned by NCBI; column B). Protein distribution in GV oocyte or cumulus cells or common to both (O: Oocyte; CC: Cumulus Cells; C: common to both; column C). Numbers of peptides and Sequest cross correlation scores (ΣXcorr) in columns D, F and E, G for oocyte and cumulus cells respectively.
(0.29 MB XLS)

Table S2 List of differentially expressed proteins in cumulus cells compared with GV oocytes. Gene name, numbers of peptides, Sequest cross correlation (ΣXcorr) score and P value for of each differentially expressed protein in GV oocyte and cumulus cells.
(0.10 MB XLS)

Table S3 Distribution of GV oocyte and cumulus cell proteins by subcellular compartments. The classification of the identified GV oocyte and cumulus proteins among subcellular compartments was performed using cellular component GO annotations.
(0.02 MB XLS)

Table S4 Proteins involved in cell-to-cell signaling biological process.
(0.04 MB XLS)
