# Supplementary figures and images for: Proteomics-Based Systems Biology Modeling of Bovine Germinal Vesicle Stage Oocyte and Cumulus Cell Interaction
Source: PLoS One. 2010 Jun 21;5(6):e11240. doi: 10.1371/journal.pone.0011240 (PMC2888582; doi:10.1371/journal.pone.0011240)

Figure S1.

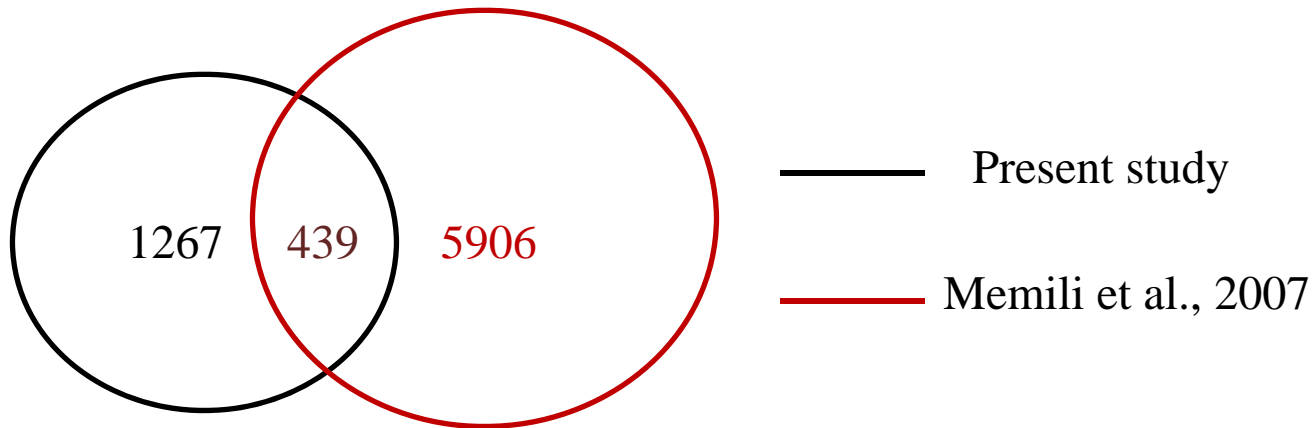

Supplement: Figure S1 — Comparison of proteins identified in present study with previous published study by Memili et al., 2007. (0.01 MB PDF) [file pone.0011240.s001.pdf]
